# *hkl*-based calculation of total scattering patterns from discrete and low-dimensional structure models using *TOPAS*

**DOI:** 10.1107/S1600576724011749

**Published:** 2025-02-01

**Authors:** Karsten Mesecke

**Affiliations:** a Leibniz Institute for Materials Engineering – IWT, Paul-Feller-Str. 1, 28199Bremen, Germany; DESY, Hamburg, Germany

**Keywords:** Rietveld method, pair distribution function, nanomaterials, modelling, simulations

## Abstract

If discrete or low-dimensional structure models are placed in large supercells in space group *P*1, the intrinsic periodicity of the Rietveld method is disrupted and their structural sites are scanned by millions of *hkl*s. This allows a Rietveld-compatible calculation of diffuse scattering and small-angle scattering which is demonstrated here for a benzene molecule, a PbS quantum dot, a hydroxy­apatite nano-fibril and turbostratic carbon. Total scattering patterns are compared with the Debye scattering equation and accompanied by composite pair distribution function modelling using the same models.

## Abstract 2

A demonstration is given of the use of discrete and low-dimensional structure models within the periodic formalism of conventional Rietveld refinement software for the calculation of total scattering patterns.

## 1. Introduction

Unravelling the atomic structure of nanomaterials at very small scales (<10 nm) is a challenging task which requires total scattering analysis using *e.g.* the Debye scattering equation (DSE) or the pair distribution function (PDF) (Bertolotti *et al.*, 2018 ▸; Cervellino *et al.*, 2010 ▸; Christiansen *et al.*, 2020 ▸; Dinnebier *et al.*, 2019 ▸; Egami & Billinge, 2012 ▸; Jensen, 2021 ▸; Thomas & Noyan, 2023 ▸). The DSE calculates a total scattering pattern (Bragg and diffuse scattering) as the sum of the scattering contribution from all the atoms and atom pairs (Debye, 1915 ▸; Cervellino *et al.*, 2010 ▸). Periodicity is not assumed, and discrete atomistic models also yield the size and shape profile in the small-angle range (Li *et al.*, 2016 ▸). The PDF is a Fourier transformation of the total scattering pattern and shows the probability of interatomic distances in the sample (Egami & Billinge, 2012 ▸; Farrow & Billinge, 2009 ▸). Transformation of experimental data requires a 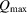 of at least 15–20 Å^−1^ and good data quality, usually obtained with designated synchrotron light sources (Jensen, 2021 ▸). The PDF of nanomaterials can be calculated from discrete atomistic models or approximated by form factor convolutions (Chen *et al.*, 2023 ▸; Christiansen *et al.*, 2020 ▸; Farrow & Billinge, 2009 ▸).

The Rietveld method (Rietveld, 1969 ▸) computes a diffraction pattern as a sum of Bragg reflections, originating from parallel lattice planes (*hkl*s), each contributing a certain intensity at a certain *Q*, *d* spacing or 2θ. It was developed in the 1960s to resolve the overlap of Bragg reflections in powder diffraction patterns based on structure models and, in order to be computationally feasible at the same time, translational periodicity was implemented (Runčevski & Brown, 2021 ▸). Periodic structure models generate only a distinct set of *hkl*s, which is perfectly suited for the analysis of microcrystalline materials or even larger nanoparticles but not for nano­materials at very small scales <10 nm (Bertolotti *et al.*, 2018 ▸; Jensen, 2021 ▸). Fortunately, the intrinsic periodicity of Rietveld-compatible structure models can be disrupted by placing them in large, otherwise empty, unit cells (Chen *et al.*, 2023 ▸; Ufer *et al.*, 2004 ▸). These supercells in space group *P*1 generate millions of *hkl*s which scan the structure model in all possible orientations and overcome the limitations of conventional periodic structure models. Applications include the analysis of turbostratically disordered phyllosilicates (Ufer *et al.*, 2004 ▸; Wang *et al.*, 2012 ▸) and nanocrystalline calcium silicate hydrates (Mesecke *et al.*, 2022 ▸). This work extends the ‘supercell approach’ to the discrete modelling of nanoparticles and even small molecules. It is demonstrated how *TOPAS* (Coelho, 2018 ▸) is capable of calculating total scattering patterns in the small-angle and wide-angle range and how the same models are suited for the composite PDF modelling recently described by Chen *et al.* (2023 ▸).

## 2. Methodology

**Figure 1:**
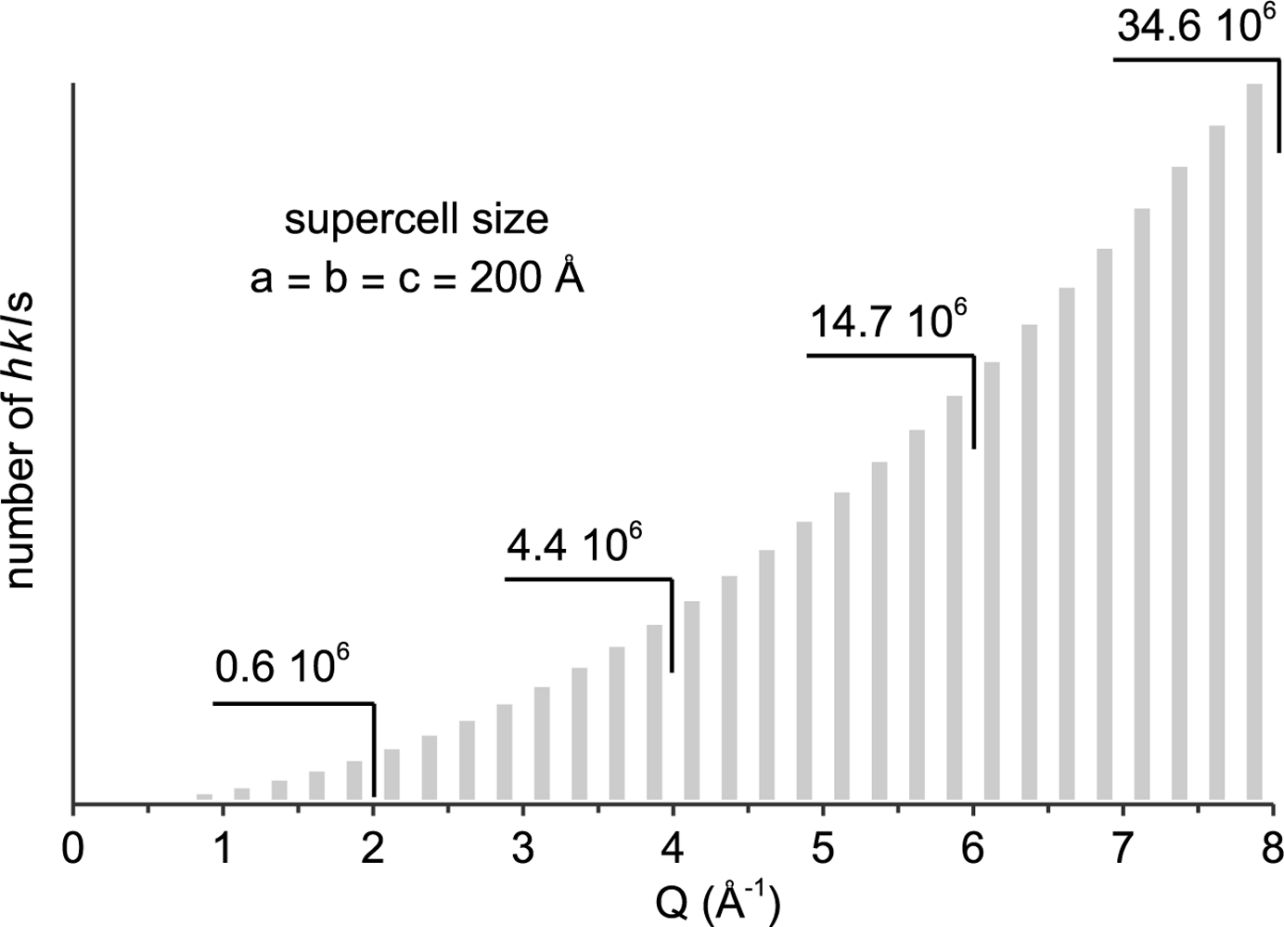
Number of *hkl*s generated by a large supercell in space group *P*1.

**Figure 2:**
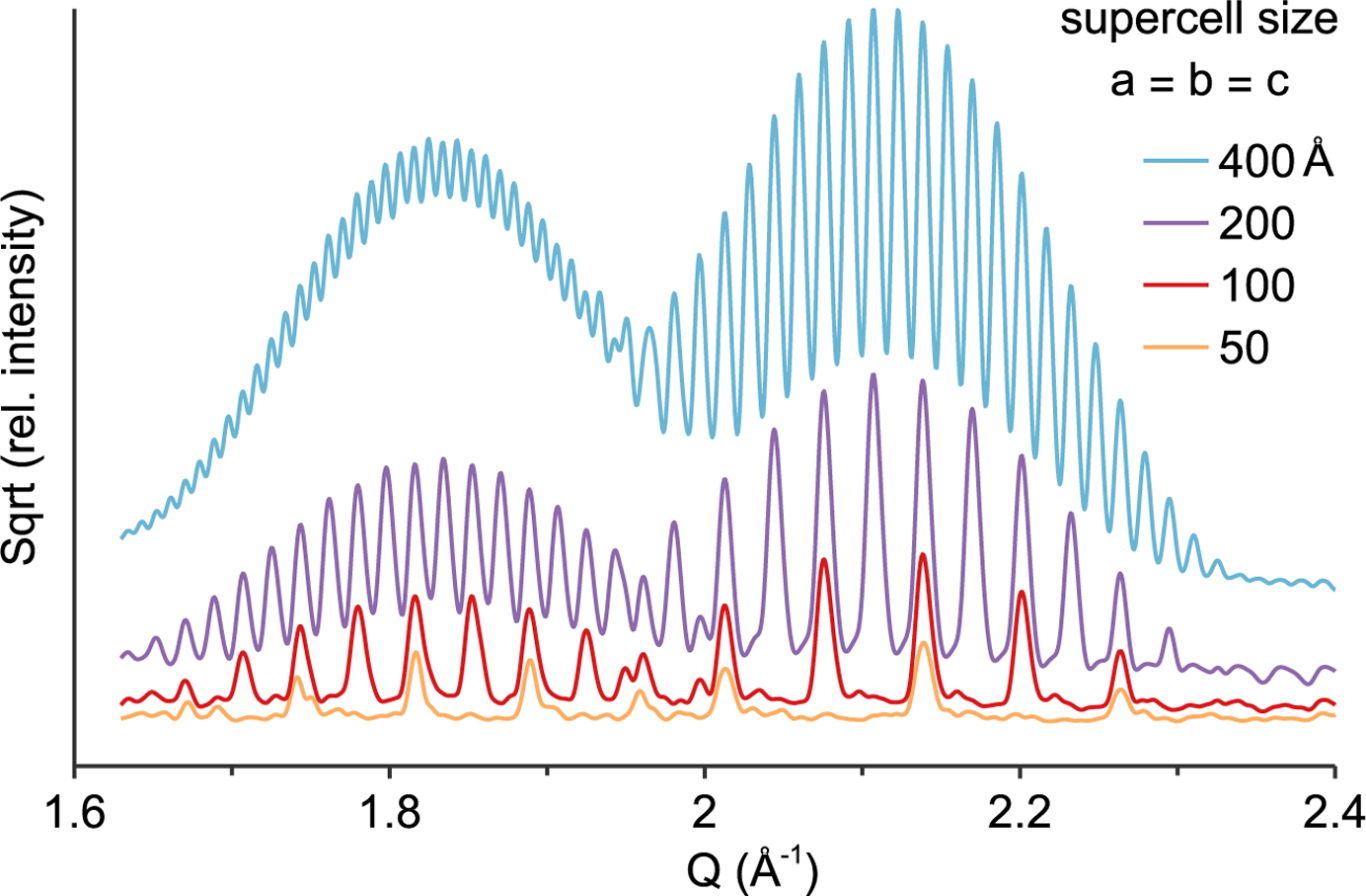
Calculated scattering patterns in relation to supercell size; 3.5 nm PbS quantum dot; Gaussian crystallite size broadening parameter value 100.

The approach described here does not alter the algorithm of the Rietveld method, it only increases the number of *hkl*s to the millions (Fig. 1 ▸). Scattering patterns of structure phases are still calculated as the sum of intensities *I* from all *hkl*s multiplied by a scale factor (Dinnebier *et al.*, 2019 ▸; Dinnebier & Scardi, 2023 ▸). Expansion of supercells in space group *P*1 increases the number of parallel lattice planes (*hkl*s) which then scan the structure model and its electron density at an increasing precision (Fig. 2 ▸). With supercell expansion, the number of *hkl*s and overall intensity increase proportionally to the supercell volume *V* (Table 1 ▸), while the structure factor and intensity of each *hkl* stay constant. In order to keep the overall intensity and mass fraction constant, the scale factor has to be decreased proportionally to the supercell volume. About 90% of *hkl*s contribute more than zero intensity *I*, whereas the top 5% of *hkl*s amount to more than 90% of the intensity (Table 1 ▸). Since the *hkl*s substantially contributing intensity are inhomogeneously distributed at demonstrated supercell sizes, broadening by a simple Gaussian crystallite size convolution is applied (Fig. 2 ▸).

Discrete or low-dimensional structure models are defined in the *TOPAS* .str structure format without the use of specific keywords or macros. It is essential to define structural sites independent of the expanded lattice parameters and supercell size by either using a rigid body or applying a formula to respective fractional coordinates. Since each fractional coordinate is intrinsically multiplied with the expanded lattice parameter, dividing it beforehand will cancel out any influence on the positions of structural sites. For discrete models all three lattice parameters are expanded, whereas for low-dimensional nanomaterials only two or one lattice parameter is expanded. The *TOPAS* .inp files for all examples can be found in the supporting information. For instance, spherical nanoparticles are defined by incrementing the original unit cell into a large cube, defining a centre and truncating all structural sites beyond the intended radius. Crystallographic information is retrieved from databases, *e.g.* the Crystallography Open Database (COD) (Gražulis *et al.*, 2009 ▸), reduced to space group *P*1 by the tool *TRANSTRU* available from the Bilbao Crystallographic Server (Aroyo *et al.*, 2011 ▸) and converted to the *TOPAS* .str structure format. The editing of many structural sites is facilitated by temporarily transferring the *TOPAS* code to a spreadsheet and breaking it up into multiple columns. Transferring the *TOPAS* code back to the *TOPAS* .str structure format is not hindered by the separation between spreadsheet cells. Structure models described in .str *TOPAS* structure format can be converted to the .xyz or .cif format, but then the supercell size is fixed.

Total scattering patterns were calculated using the Rietveld software *TOPAS Academic 8.15* (Coelho, 2018 ▸) and the DSE software *CLAUDE* from the *DEBUSSY v2.2* package (Cervellino *et al.*, 2010 ▸; Cervellino *et al.*, 2015 ▸). In both cases, a wavelength of 1.54139 Å (Cu *K*α) was used. *TOPAS* calculations apply the Lorentz–polarization factor, whereas *CLAUDE* automatically includes only the Lorentz factor and the polarization factor is added afterwards (Dinnebier & Scardi, 2023 ▸). The thermal diffuse scattering (TDS) is automatically added by *CLAUDE*, whereas *TOPAS* only attenuates the integrated intensity. Therefore, in *TOPAS* the TDS is approximated by a Chebyshev polynomial, which is refined in a separate step by adding a duplicate structure model with a scale factor multiplied by −1 and atomic displacement parameters *B*_eq_ set to 0. Computationally demanding *TOPAS* calculations with millions of *hkl*s were performed on an AMD Ryzen 5 PRO 7530U, 64-bit Windows system, with 32 GB memory.

PDF calculations, and in particular calculation of *G*(*r*), were performed using the same structure models by adding the keyword pdf_data to *TOPAS* (Coelho *et al.*, 2021 ▸). Since the average atom number density ρ_0_ of mostly empty supercells is practically zero, the sloping baseline term 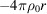 becomes ineffective. A particle shape dependent baseline is implemented using the composite modelling approach (Chen *et al.*, 2023 ▸). This approach applies a duplicate structure model with the scale factor multiplied by −1 and with very high atomic displacement parameters *B*_eq_ to smooth out individual atom–atom correlations.

## 3. Simulations

### 3.1. Benzene molecule

**Figure 3:**
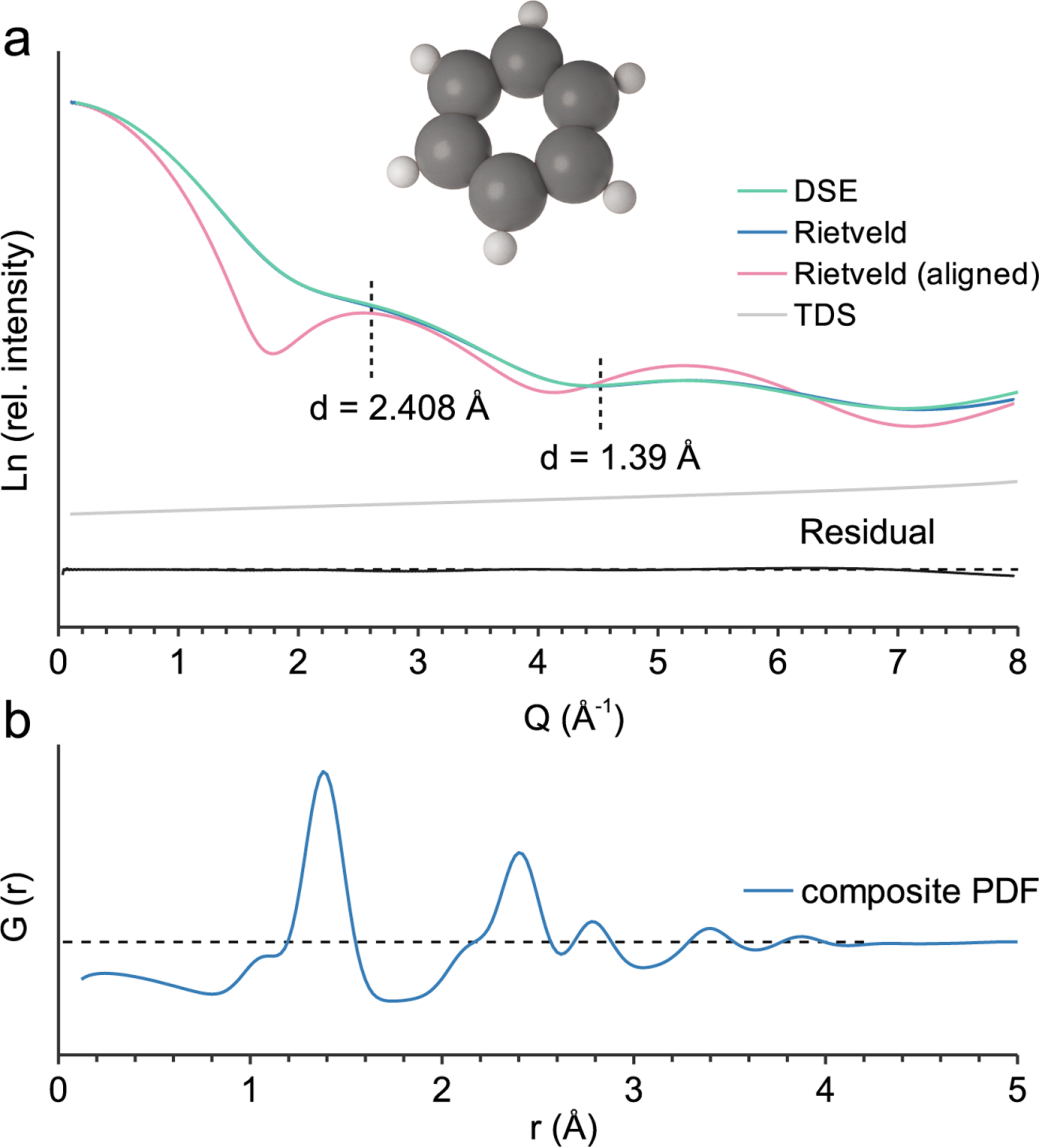
Benzene molecule. (*a*) Calculated scattering patterns without and with alignment by preferred orientation according to March–Dollase (Dollase, 1986 ▸), *a* = *b* = *c* = 200 Å, Gaussian crystallite size broadening parameter value 20; (*b*) composite PDF modelling, baseline model *B*_eq_ 10.

The smallest example is a single benzene molecule [Fig. 3 ▸(*a*)]. Thanks to advances in X-ray free-electron lasers, the scattering of individual molecules and even individual aligned molecules can be analysed in the gas phase (Kierspel *et al.*, 2019 ▸; Stankus *et al.*, 2020 ▸). The benzene model is based on the equilibrium bond lengths of Heo *et al.* (2022 ▸) with atomic displacement parameters *B*_eq_(C) 0.4 and *B*_eq_(H) 0.5. It is placed in an otherwise empty supercell *a* = *b* = *c* = 200 Å in size. The *TOPAS* calculation with an *x*-axis calculation step of 0.1 2θ takes *ca* 45 s, whereas the *CLAUDE* sampling of interatomic distances and pattern calculation are almost instantaneous. If the *TOPAS* calculation includes the approximated TDS, it almost perfectly reproduces the DSE results from *CLAUDE* [Fig. 3 ▸(*a*)]. If no preferrred orientation is implemented, calculations show only a weak maximum at *caQ* = 5.2 Å^−1^ (*d* = 1.2 Å) related to the C–C distance of 1.39 Å. In this case, the contribution from the ring diameter (*d* = 2.4 Å) is overlapping with the form factor scattering of the randomly oriented molecule. However, if the molecule is strongly aligned by preferred orientation according to March–Dollase (Dollase, 1986 ▸), a second maximum related to the carbon six-ring diameter appears at *caQ* = 2.6 Å^−1^. More obvious are the atom–atom correlations in the *G*(*r*) [Fig. 3 ▸(*b*)]. Peak shapes are severely affected here by atomic displacement parameters (Dinnebier *et al.*, 2019 ▸). The atomic dis­place­ment parameters for the baseline model are increased to *B*_eq_ 10. For comparison, the reader is referred to the benzene radial distribution function reported by Terban & Billinge (2022 ▸).

### 3.2. PbS quantum dot

**Figure 4:**
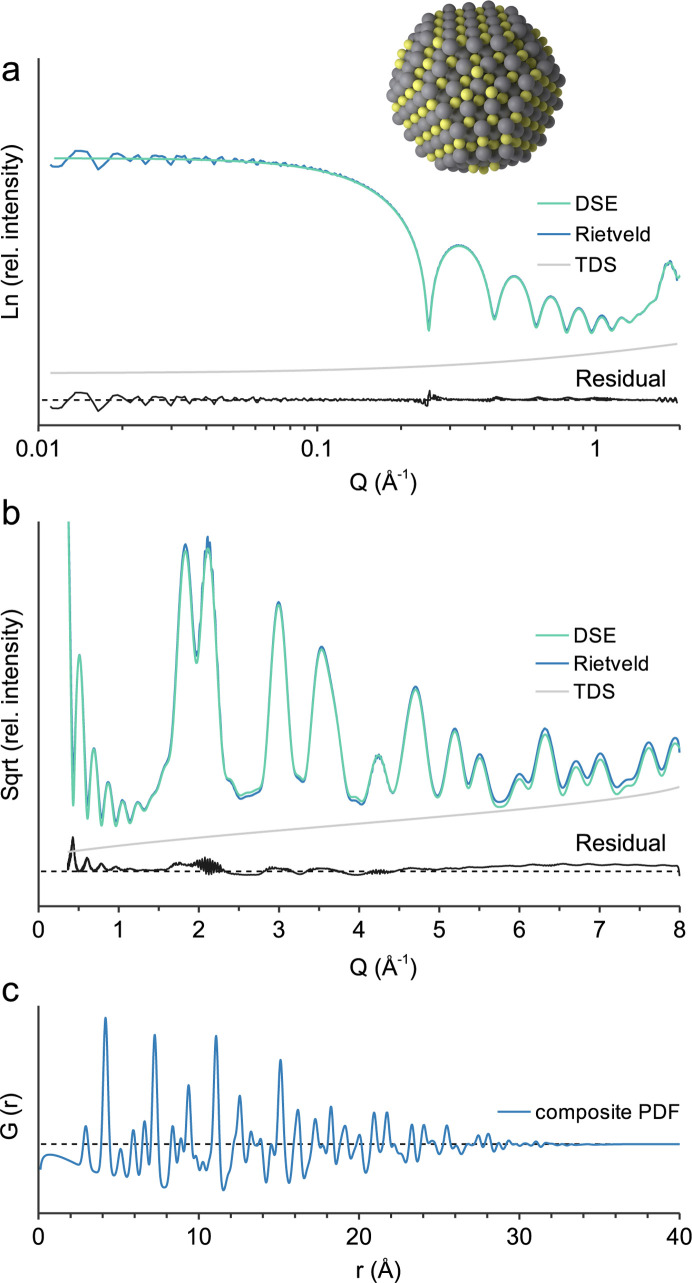
3.5 nm PbS quantum dot. (*a*) Calculated small-angle scattering; *a* = *b* = *c* = 1000 Å, no crystallite size broadening; (*b*) wide-angle scattering, *a* = *b* = *c* = 200 Å, Gaussian crystallite size broadening parameter value 20; (*c*) composite PDF modelling, baseline model *B*_eq_ 80.

The nanoparticle example is a PbS quantum dot, 3.5 nm in diameter, based on the crystal structure of galena (Noda *et al.*, 1987 ▸; COD 5000087) with 895 atoms at individually described structural sites. The supercell size is chosen in regards to the *Q* range since the number of generated *hkl*s strongly depends on *Q* (Fig. 1 ▸). In the small-angle range at low *Q* [Fig. 4 ▸(*a*)], a supercell size of *a* = *b* = *c* = 1000 Å is necessary to reduce the noise at low *Q* and reproduce the DSE calculation. The form factor oscillations are due to the uniform size of the simulated nanoparticle, which also assumes a random orientation in dilute solution without intermolecular interaction (Li *et al.*, 2016 ▸). The small-angle X-ray scattering (SAXS) of actual polydisperse samples has to be accounted for by multiple discrete models. In the wide-angle range at high *Q* [Fig. 4 ▸(*b*)] the supercell size imposes a computational burden and it is reduced to *a* = *b* = *c* = 200 Å. The *TOPAS* calculation with an *x*-axis calculation step of 0.1 2θ takes *ca* 145 s, whereas the *CLAUDE* calculations take only a few seconds. The obtained pattern [Fig. 4 ▸(*b*)] is very similar to the DSE calculation with some mismatch due to the approximation of TDS. Calculation of *G*(*r*) [Fig. 4 ▸(*c*)] is performed with *B*_eq_(Pb) 0.88 and *B*_eq_(S) 0.89, whereas for the baseline model they are increased to *B*_eq_ 80. Interestingly, *G*(*r*) seems to attenuate at a smaller diameter than the actual nanoparticle size, which is due to the small number of atoms located >3 nm apart from each other.

### 3.3. Hydroxyapatite nano-fibril

**Figure 5:**
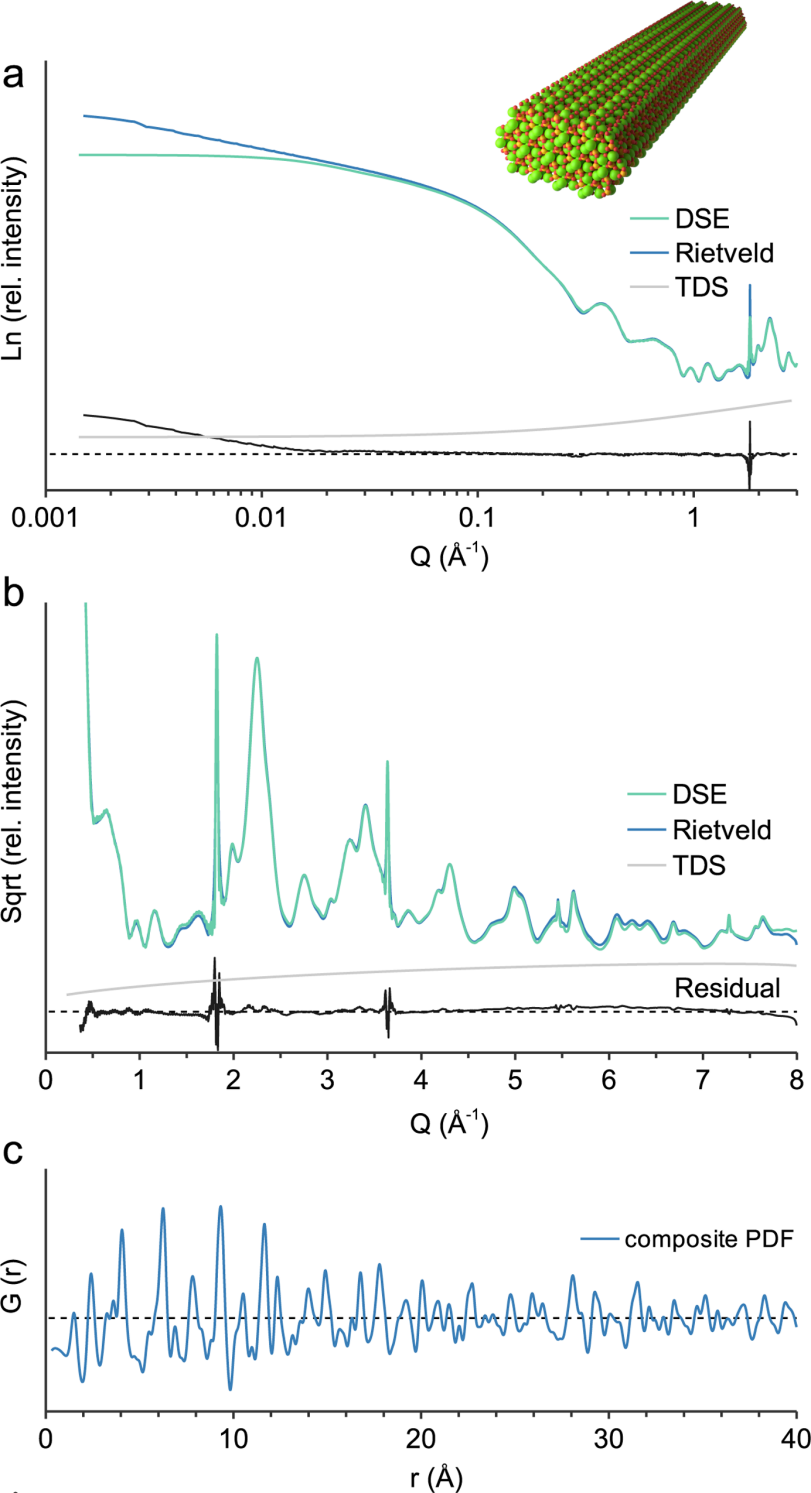
Hydroxyapatite nano-fibril with a cross section of 2.3 × 4.6 nm. (*a*) Calculated small-angle scattering, *a* = *b* = 5000 Å, no crystallite size broadening; (*b*) wide-angle scattering, *a* = *b* = 500 Å, Lorentzian crystallite size convolution parameter value 39.3; (*c*) composite PDF modelling, baseline model *B*_eq_ 30.

Low-dimensional nanomaterials with a high aspect ratio in one dimension are demonstrated here by a hydroxyapatite nano-fibril as found in bone (Bertolotti *et al.*, 2021 ▸). The *TOPAS* structure model remains periodic in one dimension and resembles a cross section of 2.3 × 4.6 nm based on the crystal structure of hydroxyapatite (Arnold *et al.*, 2022 ▸; COD 2022591). For the small-angle range [Fig. 5 ▸(*a*)] *a* and *b* are expanded to 5000 Å to sufficiently isolate the low-dimensional structure model. The remaining periodicity in the *c* dimension leads to a slope at low *Q*. In contrast, the DSE calculation by *CLAUDE* uses a discrete model (2.3 × 4.6 × 25 nm) based on the same crystal structure and cross section with 19584 structural sites, which leads to a constant scattering intensity at low *Q*. The mismatch at *Q* = 1.8 Å^−1^ is due to the lack of crystallite size broadening, which is not applicable in the small-angle range. For the wide-angle range [Fig. 5 ▸(*b*)] *a* and *b* are reduced to 500 Å. The length of the nano-fibril in the *c* dimension is accounted for by a Lorentzian crystallite size convolution with a parameter value 39.3 which corresponds to an integral breadth crystallite size of 25 nm. This leads to slightly broader 00*l* peaks at 1.8 and 3.6 Å^−1^ compared with the discrete model with one distinct length. The different nature of the structure models also leads to significantly different calculation times. Whereas the *TOPAS* calculation with an *x*-axis calculation step of 0.1 2θ takes *ca* 20 s, the *CLAUDE* sampling of interatomic distances between 19584 structural sites takes 20 min. Actual samples may be affected by *e.g.* an amorphous surface, carbonate substitution and the assembly of nano-fibrils (Bertolotti *et al.*, 2021 ▸). Calculation of *G*(*r*) [Fig. 5 ▸(*c*)] is performed with *B*_eq_(Ca) 0.62, *B*_eq_(P) 0.49 and *B*_eq_(O) 1.48, whereas the baseline model applies *B*_eq_ 30.

### 3.4. Graphene and turbostratic carbon

Graphene and turbostratic carbon are good examples of low-dimensional layered nanomaterials. They are well ordered in two dimensions whereas the layer stacking is random or absent. Structural sites for graphene are derived from graphite (Trucano & Chen, 1975 ▸; COD 9011577). The *TOPAS* structure model for one graphene layer comprises only the two original sites and remains periodic in the *a* and *b* dimensions, while *c* is expanded to 2 × 10^4^ Å. Calculation of the full scattering pattern with an *x*-axis calculation step of 0.1 2θ takes 40 s. In contrast, the DSE calculation by *CLAUDE* uses a discrete round-edged graphene sheet of 25 × 25 nm with 23683 structural sites. The sampling of interatomic distances for this one layer already takes 30 min and therefore implementation of turbostratic disorder is not attempted for discrete models. *TOPAS* multi-layer models for the implementation of turbostratic disorder and finite stack size in the *c* dimension are described here without the keywords for defective lamellar structures (Coelho *et al.*, 2016 ▸; Wang *et al.*, 2012 ▸). Random shifts along **a** and **b** are implemented by the addition of vectors generated in a spreadsheet and in order to provide many random shifts models comprise 100 or 250 layers separated by at least 8 × 10^3^ Å in very large supercells (*c* = 4 × 10^5^ Å). Although rotations are more effective at describing turbostratic disorder (Ufer *et al.*, 2004 ▸), their implementation is not possible with the remaining periodicity along **a** and **b**.

**Figure 6:**
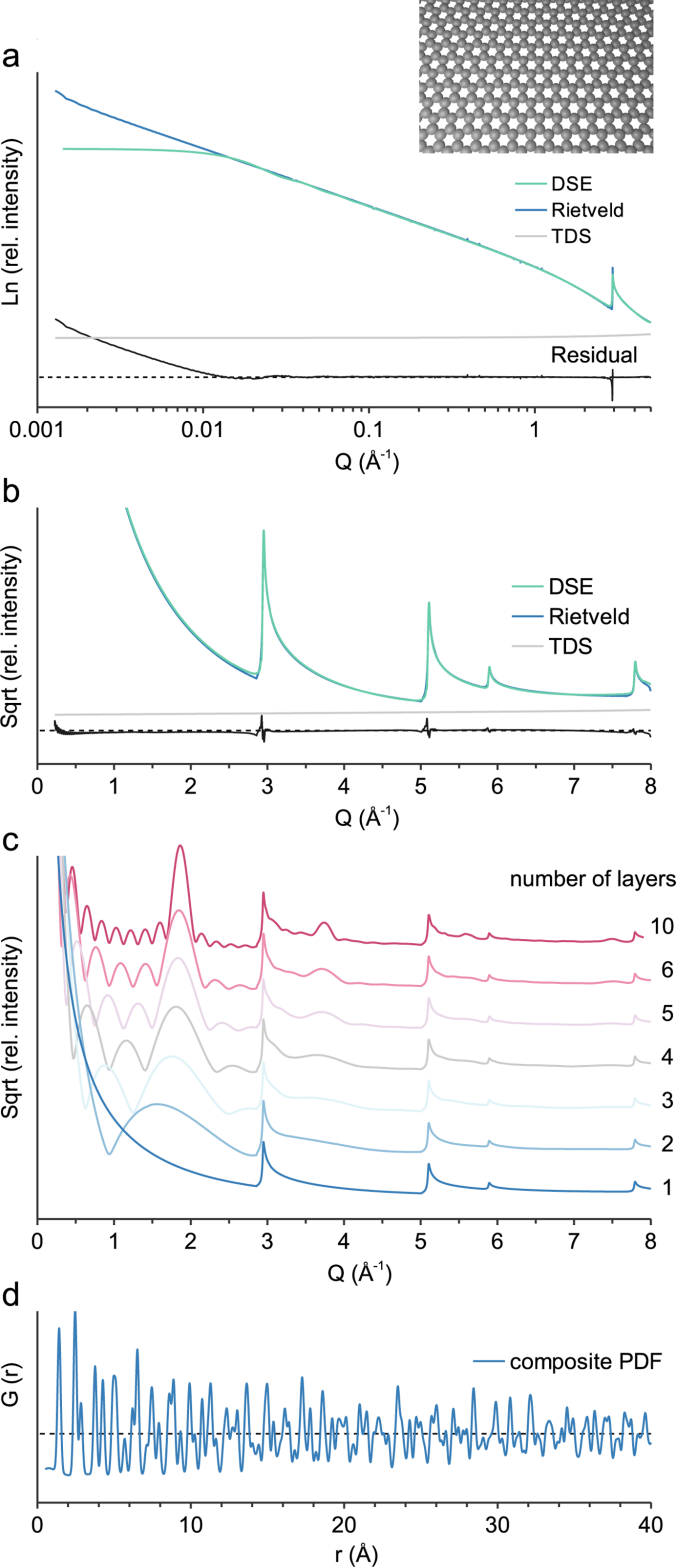
Graphene. (*a*) Calculated small-angle scattering, *c* = 2 × 10^4^ Å, no crystallite size broadening; (*b*) wide-angle scattering, *c* = 2 × 10^4^ Å, Lorentzian crystallite size convolution parameter value 39.3; (*c*) layer stacking with turbostratic disorder; (*d*) composite PDF modelling, baseline model *B*_eq_ 30.

The different nature of the structure models is observable at very low *Q* [Fig. 6 ▸(*a*)]. For the wide-angle range *TOPAS* applies a Lorentzian crystallite size convolution using a parameter value of 39.3 which corresponds to an integral breadth crystallite size of 25 nm. Calculations for the single-layer model [Fig. 6 ▸(*b*)] already show the asymmetric peaks, characteristic of turbostratic disorder, whereas multi-layer models [Fig. 6 ▸(*c*)] show the correlation between stack size and 00*l* reflections, as described by Thomas & Noyan (2023 ▸). For the comparison with actual turbostratic carbon it should be noted that form factor oscillations average out due to polydisperse stack sizes, microporosity, interstratification and the bending of layers (Saurel *et al.*, 2019 ▸). Calculation of *G*(*r*) [Fig. 6 ▸(*d*)] is performed with *B*_eq_(C) 0.4 and a baseline model with *B*_eq_ 30. For comparison the reader is referred to the work of Chen *et al.* (2023 ▸).

## 4. Conclusions

Rietveld refinement software like *TOPAS* is capable of cal­culating total scattering patterns if discrete or low-dimensional models are used. The diffuse scattering due to nanocrystallinity in the wide-angle range and the form factor scattering in the small-angle range can be calculated via *hkl*s. The presumed limitations of Bragg’s law and the Rietveld method (Bertolotti *et al.*, 2018 ▸; Dinnebier *et al.*, 2019 ▸) are just limitations of conventional periodic models with distinct sets of *hkl*s. Differences between methods arise from a different implementation of TDS and crystallite size broadening.

## 5. Outlook

The refinement of low-dimensional models was demonstrated in a previous study (Mesecke *et al.*, 2022 ▸) and it is expected to work in the same way with fully discrete models, assuming parameters are sufficiently constrained. Variations in size and shape have to be accounted for by several models. With *TOPAS* it is possible to combine discrete and conventional periodic structure models. However, the computational demand for large discrete models could be problematic, and the size limitation for nanoparticles is expected to be *ca* 8 nm. Although larger particles could be accounted for by conventional periodic models with crystallite size convolutions, investigations of particle size distributions are clearly the domain of *DEBUSSY*. The advantage of this *hkl*-based calculation is low-dimensional models, which can be much smaller then discrete models. In any case the remaining challenge is to refine large atomistic models in space group *P*1 and to implement, *e.g.*, crystallite size, crystallite anisotropy, lattice defects, surface relaxation, amorphous surface layers, strain or compositional gradients (Bertolotti *et al.*, 2018 ▸). Here the versatile scripting language, the parallel refinement of scattering patterns and *G*(*r*), and the rigid-body editor of *TOPAS* could be useful (Coelho *et al.*, 2021 ▸).

In terms of quantitative analysis, this method does not require an external or internal standard (Mesecke *et al.*, 2022 ▸; Ufer *et al.*, 2004 ▸; Wang *et al.*, 2012 ▸). It could be an alternative to the quantification of phases with partial or no known crystal structures (PONKCS) approach (Scarlett & Madsen, 2006 ▸). Discrete or low-dimensional structure models can be added to a conventional Rietveld refinement without any nanocrystallography knowledge and already approximate structure models yield reasonable quantitative results (Mesecke *et al.*, 2022 ▸). Specimen absorption correction is based on arbitrary parameter values (Coelho, 2018 ▸) and not affected by non-physical phase densities and linear mass absorption coefficients. Brindley microabsorption correction is not suited for nanocrystalline materials in general and should not be applied to discrete or low-dimensional structure models (Coelho, 2018 ▸; Madsen *et al.*, 2019 ▸). Since the diffuse scattering interferes with the background, low Chebyshev polynomials should be used.

## Supplementary Material

The structure models used for simulations: https://doi.org/10.5281/zenodo.8169025

Revised TOPAS input files for the simulation of WAXS, SAXS and PDF: https://doi.org/10.5281/zenodo.13898611

## Figures and Tables

**Table 1:** Influence of the supercell size on parameters and *hkl*s for the 3.5 nm PbS quantum dot example in the *Q* range 1.626–2.397 Å^−1^

*a*, *b*, *c* (Å)	*V* (nm^3^)	ρ (g cm^−3^)	μ (cm^−1^)	*n*_*hkl*_	Relative increase	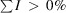 *n*_*hkl*_%	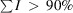 *n*_*hkl*_%
50	125	1.465	306.3	11071		87.14	4.43
100	1000	0.183	38.2	91782	8.2903	89.80	4.66
200	8000	0.023	4.8	732899	7.9852	90.10	4.59
400	64000	0.003	0.6	5864572	8.0019	89.98	4.57
